# Evaluation of physiological severity scores for predicting COVID-19 disease progression: a retrospective study

**DOI:** 10.1186/s12879-025-11127-7

**Published:** 2025-05-26

**Authors:** Sujaree Poopipatpab, Ratchaya Weerayutwattana, Pruchwilai Nuchpramool, Piyarat Phairatwet, Tospon Lertwattanachai, Konlawij Trongtrakul

**Affiliations:** 1 Department of Anesthesiology, Faculty of Medicine Vajira Hospital, Navamindradhiraj University, Bangkok, Thailand; 2 Department of Medicine, Faculty of Medicine Vajira Hospital, Navamindradhiraj University, Bangkok, Thailand; 3 Department of Pharmacology, Faculty of Medicine Vajira Hospital, Navamindradhiraj University, Bangkok, Thailand; 4 Department of Internal Medicine, Faculty of Medicine, Chiang Mai University, Chiang Mai, Thailand

**Keywords:** Non-critically ill COVID-19, Physiological severity score, Early determination, National Early Warning Score 2 Plus (NEWS2 Plus)

## Abstract

**Background:**

The coronavirus disease (COVID-19) pandemic indeed strains healthcare systems worldwide, resulting in a surge of patients with severe conditions. Numerous physiological severity scores have been assigned to assess critical conditions; however, a comprehensive comparison of these scoring systems remains lacking. Therefore, this study aimed to evaluate the performance of the severity scores upon admission in predicting the progression of COVID-19 patients to a severe condition within 14 days after hospitalization.

**Methods:**

Non-critically ill COVID-19 patients admitted to the Faculty of Medicine, Vajira Hospital, between 1 January 2021 and 30 June 2021, were assessed. We compared the discriminated ability of physiological severity scores in predicting disease progression to critical conditions using the area under the receiver operating characteristic curve (AUC).

**Results:**

Totally, 348 non-critically ill COVID-19 patients were included. Of these, 60 patients (17.2%) progressed to severe conditions within 14 days after hospitalization. The National Early Warning Score 2 with age and body mass index (NEWS2 Plus) was the most outperformed than the National Early Warning Score (NEWS), the National Early Warning Score 2 (NEWS2), the Hamilton Early Warning Score (HEWS), the Modified Early Warning Score (MEWS), and the quick Sepsis-related Organ Failure Assessment (qSOFA) scores, for predicting deterioration to severe conditions [AUC 0.77 (95% CI; 0.72-0.83)]. The NEWS2 Plus with a cutoff point of five exhibited high sensitivity (83.3%) and high negative predictive value (NPV) of 94.7%.

**Conclusions:**

NEWS2 Plus score can enhance its utility for triage of COVID-19 patients’ clinical status upon admission and guide appropriate management decisions for resource allocation.

**Trial registration:**

TCTR20241026001, registered on 26 October 2024.

**Supplementary Information:**

The online version contains supplementary material available at 10.1186/s12879-025-11127-7.

## Background

The pandemic of the coronavirus disease 2019 (COVID-19) has led to an increased number of patients facing severe or critical conditions, creating challenges for many countries due to resource limitations [1]. Approximately 20% of COVID-19 patients progress to a severe condition requiring close monitoring and specialized supportive devices, such as invasive mechanical ventilator [2]. Of these, 5% of COVID-19 patients progress to critical conditions, including respiratory failure, septic shock, and multiple organ dysfunction [3]. Early identification of high-risk COVID-19 patients is crucial for preventing further complication and reducing mortality.

Risk assessment tools, including clinical scoring systems and predictive models, assist healthcare providers in effective triage these patients for a better care [4, 5]. Physiological severity scores, such as the National Early Warning Score (NEWS) and the Sequential Organ Failure Assessment (SOFA) score, are routinely used to assess acute illness severity [6, 7]. These scores incorporate easily measurable, including body temperature (BT), respiratory rate (RR), systolic blood pressure (SBP), heart rate (HR), oxygen saturation (SpO2), oxygen supplement, and level of consciousness.

During the COVID-19 pandemic, numerous studies demonstrated the efficacy of these scores in predicting progression to critical conditions requiring intensive care unit (ICU) admission [8–12]. Various physiological severity scores, including the NEWS, a modified version of the National Early Warning Score for COVID-19 Infected Patient (NEWS-C) [13], National Early Warning Score 2 (NEWS2) [5], National Early Warning Score 2 with age and body mass index (NEWS2 Plus) [14], Hamilton Early Warning Score (HEWS) [15], Modified Early Warning Score (MEWS) [10], and quick Sepsis-related Organ Failure Assessment (qSOFA) [10, 11, 16] have been utilized to evaluate disease severity, ICU requirements, and mortality over difference timeframes.

However, studies specifically examining COVID-19 disease progression within 14 days after admission remain limited. Insights from such studies could enhance clinical decision-making, optimize resource allocation, and enable timely therapeutic interventions. The primary aim of our study is to evaluate the accuracy performance of various physiological severity scores at admission to identify non-critically ill COVID-19 patients at risk for progression to critical illness within 14 days of hospitalization. Secondary objectives include evaluating the length of hospital stay (LOS), 14-day mortality, and overall hospital mortality.

## Methods

### Study design and participants

This retrospective study enrolled COVID-19 patients admitted to the Faculty of Medicine Vajira Hospital, Navamindradhiraj University, Bangkok, Thailand, between 1 January 2021 and 30 June 2021. The study was approved by the Vajira Institutional Review Board (approval number COA 167/2564 on July 19, 2021), and had been registered on ClinicalTrials.gov (TCTR20241026001). The study was conducted in accordance with the ethical principle of the Declaration of Helsinki for medical research involving human subjects. The patient’s informed consent was waived due to minimal risk, and data were extracted from medical records and analyzed anonymously.

During COVID-19 pandemic, symptomatic patients under investigation for COVID-19 at our institution were admitted for isolation upon diagnosis, following the clinical practice guidelines established by the Department of Medical Services [17]. Critically ill patients received care in intensive care units (ICUs), while non-critically ill patients were managed in cohort wards. Data for COVID-19 patients were retrieved using the ICD-10 version 2019 code U07.1, assigned to laboratory-confirmed COVID-19 cases regardless of clinical severity. Inclusion criteria included patients aged equal 18 years or older with COVID-19 confirmed by a positive reverse transcription-polymerase chain reaction (RT-PCR) from nasal secretions. with non-critically ill COVID-19 at the time of admission. Exclusion criteria included patients aged under 18 years, patients with an artificial airway, critically ill patients - defined as a score of five to nine on the WHO Working Group on the Clinical Characterization and Management of COVID19 infection ordinary scale [18], which included acute respiratory distress syndrome (ARDS), sepsis/septic shock, and requirement of life-sustaining treatments such as mechanical ventilation, vasopressor therapy, or extra-corporeal membrane oxygenation (ECMO) [19], and hospital-acquired COVID-19 infections - defined as patients who tested positive for RT-PCR for COVID-19 post-admission during hospitalization.

### Data collection and definitions

We collected data from non-critically ill COVID-19 patients, defined as those scoring one to four on the World Health Organization (WHO) ordinary scale [18] at admission. Collected data included demographic data, pre-existing comorbidities, and vital signs. Key physiological parameters recorded at admission comprised BT, RR, SBP, diastolic blood pressure (DBP), mean arterial blood pressure (MAP), HR, and SpO_2_. Additional data included age, body mass index (BMI), oxygen supplementation status, and level of consciousness.

These parameters were utilized to calculate various physiological severity scores, including HEWS, MEWS, NEWS, NEWS-C, NEWS2, NEWS2 Plus, and qSOFA, as shown in Table 1 and Supplementary Table 1.

**Table 1:** Physiological severity scores

Parameters	NEWS	NEWS-C	NEWS2	NEWS2Plus	HEWS	MEWS	qSOFA
Age (years)		√		√			
Body mass index (kg/m^2^)				√			
Consciousness level	√	√	√	√	√	√	√
Body temperature (°C)	√	√	√	√	√	√	
Respiratory rate (bpm)	√	√	√	√	√	√	√
Systolic blood pressure (mmHg)	√	√	√	√	√	√	√
Heart rate (bpm)	√	√	√	√	√	√	
SpO2 (%)	√	√	√	√	√		
Oxygen supplement	√	√	√	√	√		

*Abbreviations*: *HEWS* Hamilton Early Warning Score, *MEWS* Modified Early Warning Score, *NEWS* National Early Warning Score, *NEWS-C* A Modified version of the National Early Warning Score for COVID-19 Infected Patient, *NEWS2* National Early Warning Score 2, *NEWS2 Plus* National Early Warning Score 2 with age and body mass index, and *qSOFA* quick Sepsis-related Organ Failure Assessment

NEWS-C and NEWS2 Plus were modified versions of NEWS and NEWS2, respectively, incorporating additional parameters-age and BMI. In NEWS-C, zero points were assigned to patients aged < 65 years, while three points were assigned to those aged ≥65 years [13]. For NEWS2 Plus, age was categorized as follows: zero points for individuals aged < 40 years, three points for those aged 40–59 years, and four points for those aged ≥60 years. BMI was scored as follows: zero points for BMI < 24.9 kg/m^2^, two points for BMI between 25.0–29.9 kg/m^2^, and three points for BMI ≥ 30 kg/m^2^ [14], as shown in Supplementary Table 1.

Patients were monitored for 14 days following admission to assess progression to critical COVID-19, defined as a score of five to nine on the WHO ordinary scale [18], accompanied by conditions such as ARDS, sepsis/septic shock, or the requirement of life-sustaining treatments, including mechanical ventilation, vasopressor therapy, or ECMO [19]. Other data were collected as the length of hospital stay (hosp-LOS), 14-day mortality, and overall hospital mortality.

### Sample size calculation

The sample sizes were calculated based on the accuracy defined by the area under the receiver operating characteristic curve (AUC), a critical value for a 95% confidence level of 1.96, and a precision of 0.05. The variance of AUC can be parametrically estimated using the binormal assumption or the exponential approximation according to the Hanley and McNeil formula [20]. In a study by De Socio GV et al., the AUC of NEWS2 to predict critically ill COVID-19 patients were 0.87 [21]. We adopted this AUC as the expected AUC in our study, with a null hypothesis AUC of 0.5. Therefore, the expected effect size was 0.37. Accounting for a power of 0.80 and a significant level of 0.05, a total population of 325 cases were required in our study.

### Statistical analysis

Continuous variables were presented as median and interquartile range (IQR 1, 3), while categorical variables were expressed as frequencies and percentages. The authors employed the Mann-Whitney U test to compare continuous variables and Fisher’s exact test for categorical variables.

We evaluated the performance of the physiological severity scores in predicting clinical outcomes was the progression to critical COVID-19 within 14 days after admission by using Receiver Operating Characteristic (ROC) curves. Subsequently, we calculated the AUC to demonstrate how each physiological severity score could identify COVID-19 patients with critical conditions and pair-wisely compared these scores using a DeLong test. Additionally, the cutoff points for these physiological severity scores were utilized based on previous studies to identify moderate-risk patients and guide decisions regarding escalation to the critical care team as follows: five for NEWS2 Plus [14]; five and six for HEWS [22], NEWS [23, 24], NEWS-C [13], and NEWS2 [23, 24]; three and four for MEWS [12]; and two for qSOFA [25].

Additionally, a sensitivity analysis was performed by varying the cutoff points for each physiological severity score. The Youden index was used to determine the optimal cutoff point [26] and to evaluate sensitivity, specificity, positive predictive value (PPV), negative predictive value (NPV), positive likelihood ratio (LR+), and negative likelihood ratio (LR-). Statistical analysis for this study was conducted using the established computer program Stata/MP Version 16.1 from Stata Corp, College Station, TX, USA.

## Results

### Patients’ baseline characteristics

**Fig. 1:**
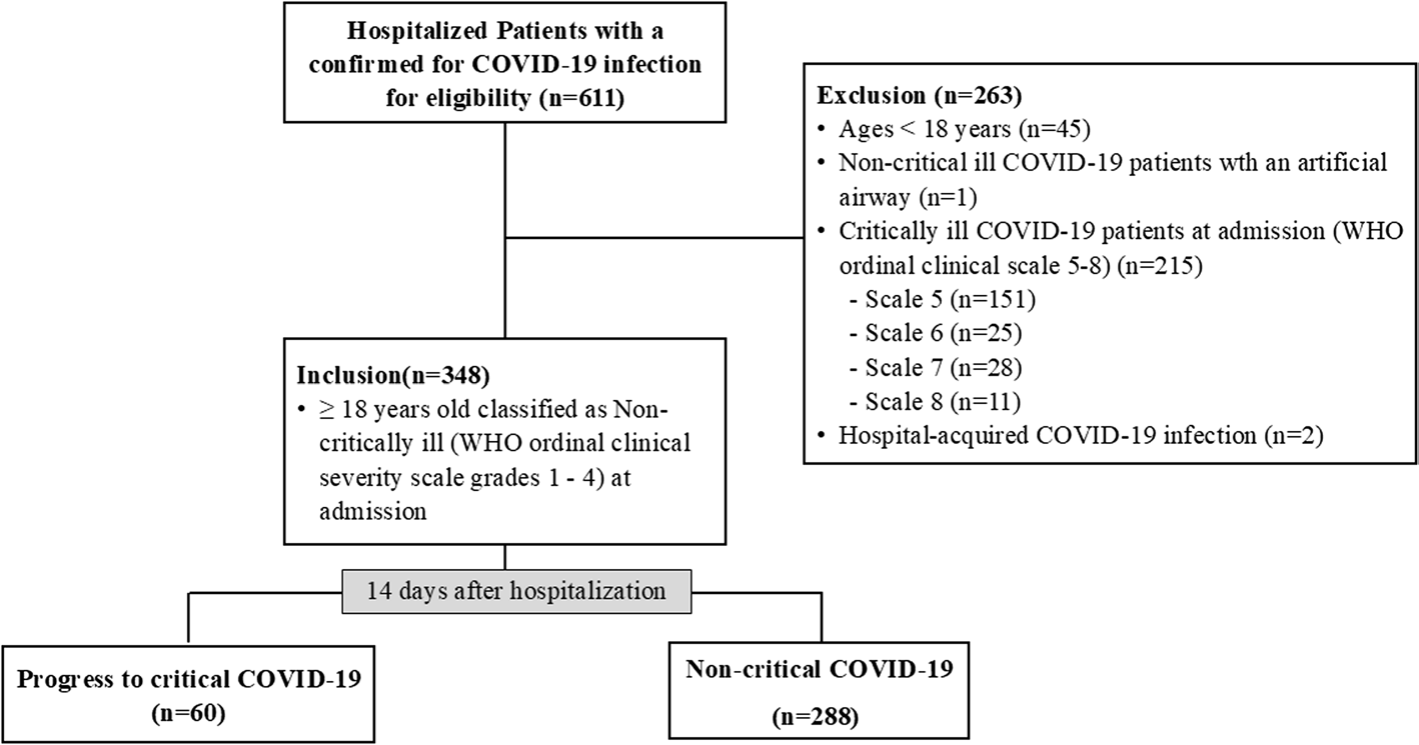
The study flow diagram

A total of 348 non-critically ill COVID-19 patients were included in this study. These patients were in the early phase of SAR-CoV-2 infection, either asymptomatic or presenting with mild symptoms upon admission. Among them, 347 had a Glasgow Coma Scale (GCS) of 15, while one had a GCS of 14. The median time from symptoms onset or known contact with a COVID-19-positive individual to hospital admission was three days. Of 348 patients, sixty cases (17.2%) deteriorated to critical COVID-19 within 14 days after admission, as shown in Fig. 1.

The non-critically ill COVID-19 patient’s characteristics were presented in Table 2. The median age was 48 (IQR 31, 62) years, 43.1% were male, and the median BMI was 24.2 (IQR 21.3, 27.3) kg/m^2^. The baseline HEWS, MEWS, NEWS, NEWS-C, NEWS2, NEWS2 Plus, and qSOFA at admission were 0 (IQR 0, 1), 1 (IQR 1, 2), 1 (IQR 0, 2), 2 (IQR 1, 3). 1 (IQR 0, 2), 4 (IQR 3, 6), and 0 (IQR 0, 0), respectively.

**Table 2:** Clinical characteristics of COVID-19 patients at hospital admission

Variables	All patients (*n*=348)	Critical group (*n*=60)	Non-critical group (*n*=288)	*p*-value
Demographics				
Age (years)	48 (31–62)	60 (52–70)	43 (29–59)	<0.001
Male, n (%)	151 (43.4)	32 (53.3)	119 (41.3)	0.12
BMI (kg/m^2^)	24.2 (21.3–27.3)	25.3 (23.5–29.6)	23.8 (20.9–27.1)	0.006
Co-morbidities, n (%)				
Diabetes	62 (17.8)	19 (31.7)	43 (14.9)	0.005
Hypertension	102 (29.3)	26 (43.3)	76 (26.4)	0.01
Dyslipidemia	60 (17.2)	20 (33.3)	40 (13.9)	0.001
Others	79 (22.7)	18 (30.0)	61 (21.2)	0.17
Onset of symptoms prior admission (day)	3 (1–5)	3.5 (2–5)	3 (1–5)	0.05
Vital signs at admission				
Alert, n (%)	348 (100)	60 (100)	288 (100)	-
Body temperature (ºC)	37.0 (36.6–37.6)	37.5 (36.8–38.1)	37.0 (36.6–37.5)	0.005
Respiratory rate (bpm)	20 (20-20)	20 (20-20)	20 (20-20)	0.09
Systolic blood pressure (mmHg)	127 (115–138)	131 (119–149)	126 (114–137)	0.06
Diastolic blood pressure (mmHg)	80 (72–88)	83 (73–92)	80 (72–87)	0.16
Mean arterial blood pressure (mmHg)	95 (88–104)	99 (91–109)	95 (87–103)	0.04
Heart rate (bpm)	90 (82–100)	91 (83–101)	90 (81–100)	0.64
Respiratory rate (bpm)	20 (20-20)	20 (20-20)	20 (20-20)	0.09
SpO2 (%)	97 (96–98)	96 (95–98)	97 (97–98)	< 0.001
The physiological severity scores				
NEWS	1 (0–2)	2 (1–4)	1 (0–2)	<0.001
NEWS-C	2 (1–3)	3 (1–5)	1 (1–3)	<0.001
NEWS2	1 (0–2)	1 (1–3)	1 (0–2)	0.01
NEWS2 Plus	4 (3–6)	6 (5–8)	4 (2–6)	<0.001
HEWS	0 (0–1)	1 (0–3)	0 (0–1)	<0.001
MEWS	1 (1–2)	2 (1–2)	1 (1–2)	0.004
qSOFA	0 (0-0)	0 (0–1)	0 (0-0)	<0.001
Outcomes within 14 days, n (%)				
HFNC	19 (5.5)	19 (31.7)	0 (0)	<0.001
IMV	12 (3.5)	12 (20.0)	0 (0)	<0.001
ARDS	50 (14.4)	50 (83.3)	0 (0)	<0.001
Septic shock	52 (14.9)	52 (86.7)	0 (0)	<0.001
Usage of vasopressor	5 (1.4)	5 (8.3)	0 (0)	<0.001
ICU admission	55 (15.8)	20 (33.3)	35 (12.2)	<0.001
Length of hospital stay (days)	8 (5–11)	12 (8–18)	7 (4–10)	<0.001
14-day mortality	1 (0.3)	1 (1.7)	0 (0)	<0.17
Hospital Mortality	6 (1.7)	6 (10.0)	0 (0)	<0.001

Continuous data are presented as median (interquartile range)

*Abbreviations*: *BMI* Body mass index, *HFNC* high flow nasal cannula, *HEWS* Hamilton Early Warning Score, *MEWS* Modified Early Warning Score, *NEWS* National Early Warning Score, *NEWS-C* A Modified version of the National Early Warning Score for COVID-19 Infected Patient, *NEWS2* National Early Warning Score 2, *NEWS2 Plus* National Early Warning Score-2 with age and body mass index, and *qSOFA* and quick Sepsis-related Organ Failure Assessment

Significant differences were observed between the progress to the critical COVID-19 group and the non-critical COVID-19 group within 14 days after hospitalization regarding age, BMI, diabetes, hypertension, dyslipidemia, BT, MAP, SpO2, and various severity scoring systems including HEWS, MEWS, NEWS, NEWS-C, NEWS2, NEWS2 Plus, and qSOFA (*p*<0.05), as shown in Table 2.

The critical group had a longer median hospital stay of 12 (IQR 8,18) days compared to 7 (IQR 4,10) days in the non-critical group (*p* < 0.001). The critical group had one patient who died within 14 days and the hospital mortality rate was 1.7% [6 patients].

### Comparing physiological severity scores of non-critically ill COVID-19 patients to predict severity

**Fig. 2:**
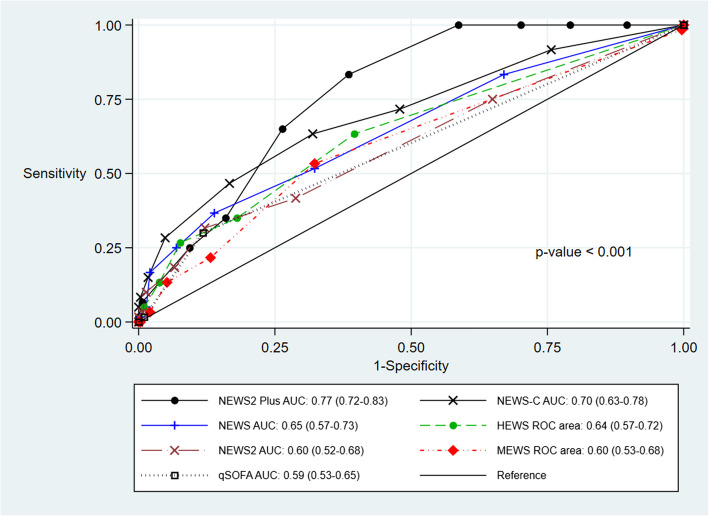
The discriminative ability of physiological severity scores in predicting COVID-19 disease progression

The results of ROC curve analysis were presented in Fig. 2 and Table 3. Among the physiological severity scores, the NEWS2 Plus demonstrated the highest accuracy in discriminating non-critically ill COVID-19 patients who were at risk of deteriorating to critically ill status within 14 days after admission [AUC 0.77 (95% CI; 0.72–0.83)]. Pairwise comparisons of the AUC values were conducted using the DeLong test, as shown in Table 4. Notably, NEWS2 Plus demonstrated statistically significant differences compared to all other scores (all *p*<0.05), except for NEW-C (*p*=0.11). In contrast, NEWS-C exhibited a significant difference when compared to qSOFA (*p*=0.047). No other pairwise comparisons demonstrated statistical significance.

**Table 3:** Performances of physiological severity score

Physiological Severity scores	Overall AUC	(95% CI)
NEWS2 Plus	0.77	(0.72–0.83)
NEWS-C	0.70	(0.63–0.78)
NEWS	0.65	(0.57–0.73)
HEWS	0.64	(0.57–0.72)
NEWS2	0.60	(0.52–0.68)
MEWS	0.60	(0.53–0.68)
qSOFA	0.59	(0.53–0.65)

**Table 4:** Comparison of AUC values of different physiological severity scores using the delong test

Score	NEWS2 Plus	NEWS-C	NEWS	HEWS	NEWS2	MEWS
NEWS-C	0.11	-	-	-	-	-
NEWS	0.002	0.16	-	-	-	-
HEWS	0.005	0.39	0.89	-	-	-
NEWS2	<0.001	0.09	0.39	0.55	-	-
MEWS	0.003	0.20	0.77	0.65	0.81	-
qSOFA	<0.001	0.047	0.29	0.48	0.78	0.64

The cutoff point for physiological severity scores from the previous studies were used to identify patients with moderate risk and to guide decisions regarding escalation of the scores to the critical care team. Among these scores as shown in Table 5, NEWS2 Plus demonstrated the highest performance in predicting the progression of disease to critical illness, with high sensitivity (83.3%), and a high negative predictive value (94.7%) at a cutoff point of five (Table 5).

**Table 5:** Comparison the current cutoff point of physiological severity scores for predicting COVID-19 disease progression

Physiological Severity scores	Cutoff point	Sensitivity (%)	Specificity (%)	PPV	NPV	+LR	-LR
NEWS2 Plus	5	83.3	61.5	31.1	94.7	2.16	0.27
NEWS-C	5	28.3	95.1	54.8	86.4	5.83	0.75
	6	15.0	98.3	64.3	84.7	8.64	0.87
NEWS	5	16.7	97.9	62.5	84.9	8.00	0.85
	6	5.0	98.6	42.9	83.3	3.60	0.96
HEWS	5	5.0	99.0	50.0	83.3	4.80	0.96
	6	0.0	99.7	0.0	82.7	0.0	1.0
NEWS2	5	10.0	98.6	60.0	84.0	7.20	0.91
	6	3.3	99.3	50.0	83.1	4.80	0.97
MEWS	3	21.7	86.8	25.5	84.2	1.64	0.90
	4	13.3	94.8	34.8	16.4	2.56	0.91
qSOFA	2	1.7	99.0	25.0	82.8	1.60	0.99

The cutoff points were from the previous studies as follows: five for NEWS2 Plus [14]; five and six for NEWS-C [13], NEWS [23, 24], HEWS [22], NEWS2 [23, 24]; three and four for MEWS [12]; and two for qSOFA [25]

Similarly, sensitivity analysis employing the Youden index to determine the optimal cutoff point demonstrated that the NEWS2 Plus at 4.5 remained the most robust predictor, with high sensitivity (83.3%), and a high negative predictive value (94.7%), as shown in Supplementary Table 2.

## Discussion

The COVID-19 pandemic presents significant challenges worldwide, particularly due to shortage of cohort ward and ICU beds, complicating patient allocation management. Several studies have demonstrated the utility of various physiological severity scores in predicting clinical deterioration, ICU need, and mortality [8–12, 21]. These scores can help clinicians allocate resources and provide proper guidance to COVID-19 treatment. We assessed the performance of physiological severity scores, including the NEWS, NEWS-C, NEWS2, NEWS2 Plus, HEWS, MEWS, and qSOFA, upon admission to identify non-critically ill COVID-19 patients at risk of deteriorating to severe COVID-19 within 14 days.

This study demonstrated that the NEWS2 Plus and NEWS-C scores exhibited moderate to good predictive performance, with the AUC of 0.77 and 0.70, respectively, and potential utility in triaging non-critically ill COVID-19 patients at risk of progression to critical illness. In contrast, the NEWS2 and others physiological severity scores, including NEWS, HEWS, MEWS, and qSOFA, showed poor performance (AUCs < 0.70).

These findings aligned with a multi-hospital study that reported a median NEWS2 of 1 (IQR 0,2), suggesting poor to moderate predictive performance in assessing COVID-19 outcomes within 14 days of admission [9]. However, several previous studies have identified the NEWS2 upon admission as a reliable predictor of ICU admission or in-hospital mortality among the COVID-19 patients during hospitalization [8, 9, 21, 27], which contrasts with our finding.

A previous study reported that the HEWS, NEWS, NEWS2, and Standard Early warning score (SEWS) generally performed well in predicting COVID-19 severity, except for MEWS [24]. The significance of NEWS2 and other physiological severity scores have been validated across different periods and patient outcomes.

MEWS and qSOFA performed poorly in identifying the COVID-19 patients at high risk of clinical deterioration to critically illness [11, 16, 28]. The dominant symptom affects the respiratory system [5]. Several studies have demonstrated physiological parameters, including SpO2, FiO2, and RR, as part of the Quick COVID-19 severity index and the ROX index (the ratio of oxygen saturation/fractional oxygen to respiratory rate), performed well in predicting clinical deterioration in the COVID-19 patients receiving HFNC [12, 29–31]. Therefore, considering SpO2 and the oxygen supplement variables in these scores may enhance their predictability for clinical deterioration in COVID-19 patients. However, MEWS and qSOFA, exclude parameters such as oxygen supplement and SpO2.

NEWS-C, which incorporated age into NEWS for COVID-19 screening, has not demonstrated significant predictive performance [13, 32]. Previous studies reported that the predictive performance of NEWS could be enhanced by integrating additional variables such as age, ICU admission, chronic disease, and laboratory blood tests (such as C-reactive protein, B-type natriuretic peptide, alanine aminotransferase, aspartate aminotransferase, lactate, creatinine and urea) [11]. Another study reported that added variables as supplemental oxygen flow rate, urea, age, oxygen saturation, CRP, estimated GFR, neutrophil count, and neutrophil-to-lymphocyte ratio to NEWS2 improved its predictive performance compared to NEWS2 alone [5].

Age and BMI enhanced the efficacy of the NEWS2 in identifying severe COVID-19 pneumonia [14]. Our study showed that the Early Warning Score, combined with other clinical data such as NEWS-C (which combined NEWS with age) and NEWS2 Plus had moderate to good performance for triaging non-critically ill COVID-19 patients to progress in severe within 14 days of hospitalization. A higher AUC reflected better discrimination, particularly in asymptomatic or mildly symptomatic patients. This improved discrimination can facilitate early risk stratification, guide timely interventions, optimize resource allocation, and potentially improve patient outcomes.

Numerous cutoff points of physiological severity scores for identifying non-COVID-19 patients with moderate risk to guide decisions regarding escalation therapy to the critical care team [12–14, 23–25]. This study utilized these cutoff points to predict the progression of non-critically ill COVID-19 patients to critical illness. At a cutoff point of five, NEWS2 Plus demonstrated high sensitivity (83.3%), and a high NPV (94.7%).

When using the Youden index to determine the optimal cutoff point from our dataset, NEWS2 Plus remained the best predictor, demonstrating high sensitivity (83.3%), and a high negative predictive value (94.7%) at a cutoff point of 4.5. Thus, our study found that NEWS2 Plus is useful for physicians in excluding patients at risk of progressing to critical COVID-19 when the NEWS2 Plus score is below five in the non-critically ill group. However, additional clinical factors should be considered before applying this score in routine clinical practice.

### Strengths

The pathogenesis and manifestations of COVID-19 can be divided into two phases. The early phase is characterized by viral loads of SARS-CoV-2, during which patients are either asymptomatic or presented with mild symptoms [33]. The timeline of disease progression in mild and severe COVID-19 cases appears to correlate with viral activity and clinical manifestation [34].

Nowadays, the COVID-19 Treatment Guidelines Panel, as per the National Institutes of Health, recommends that hospitalized patients who do not require oxygen therapy but are at high risk of progressing to severe COVID-19 should initiate treatment with Ritonavir-boosted nirmatrelvir within 5 days of symptom onset, Remdesivir within 7 days of symptom onset, or Molnupiravir within 5 days of symptom onset or as soon as possible [35].

Our study population focused on non-critically ill COVID-19 patients upon admission. The median time from symptoms onset or known contact with a COVID-19-positive individual to hospital admission was three days. The physiological severity scores of both groups were low upon admission, with most clinical patients in the mild zone. However, the critical group had a significantly higher score than the non-critical group.

At the cutoff point of five, NEWS2 Plus was a good performance predictor (AUC 0.72) and demonstrated high sensitivity (83.3%) and a high NPV (94.7%). This score, which included physiological parameters, was easily recorded and promptly calculated, thereby enabling early screening of non-critically ill COVID-19 patients at risk for critical illness within 14 days of hospitalization. Moreover, age and BMI, as key predictors of severe COVID-19, provide added value in predicting disease severity.

### Limitations

There were some limitations to our study. First, this study was a single center with a small sample size, limited to a population. Second, the study was a retrospective study, the data were collected only during the first 14 days of hospitalization without follow-up on clinical outcomes beyond this period. Third, this evaluated the total score of physiological severity scoring systems rather than individual parameters. Early warning system flag high risk patients based on total scores or individual parameters exceeding three. In COVID-19, which primarily affects the respiratory system, patients may exhibit “happy hypoxemia” [23], appearing clinically stable despite significant oxygenation deficits. Our study demonstrated that patients with low scores can still present with severe conditions, underscoring the importance of close monitoring. Moreover, although NEWS2 Plus demonstrated the highest AUC, the difference in clinical significance compared to conventional scores, such as NEWS2, appears minimal. Adding age and BMI to NEWS2 may not yield substantial clinical improvement. However, given the physiological impact of aging and obesity – characterized by an increased vulnerability to organ failure due to an impaired cardiorespiratory reserve and dysregulated immune function – NEWS2 Plus should enhance the prediction of COVID-19 progression compared to other scores. To validate the clinical utility of NEWS2 Plus, prospective multi-center studies are needed to assess its impact on reducing adverse ICU outcomes.

Fourth, our study undermines vaccination information, which is an independent factor in the severity illness. Finally, like other RNA viruses, SARS-CoV-2 is constantly evolving through random mutations. which can potentially increase or decrease its infectiousness and virulence. Additionally, this study was conducted before the emergence of the SARS-CoV-2 Omicron strain, which is characterized by shorter symptom duration, lower hospitalization rates, and minimal requirement for supplement oxygen [36], partially due to lower replication efficiency in the lung parenchyma compared to other SARS-CoV-2 variants [37]. These distinct clinical and virological characteristics may result in lower physiological scores than other variants, potentially affecting predictive accuracy. The authors acknowledge the limitation regarding the lack of information on the Omicron strain in this study and accept the fact that further research focusing on this variant would be highly valuable and beneficial to the field.

## Conclusion

NEWS2 combined with age and BMI (NEWS2 Plus score) can enhance its utility for triage of non-critically ill COVID-19 patients upon admission who may deteriorate critically ill, thereby guiding appropriate patient allocation and management. However, these scores alone may not be sufficient to confirm that the patient will become critical, further examination and following clinical are necessary for appropriate care.

## Supplementary Information

Supplementary Material 1.

## Data Availability

The datasets used and/or analyzed during the current study are available from the corresponding author on reasonable request.

## Abbreviations

BT: Body temperature
RR: Respiratory rate
SBP: Systolic blood pressure
HR: Heart rate
SpO2: Oxygen saturation
BMI: Body mass index
HFNC: High flow nasal cannula
HEWS: Hamilton Early Warning Score
MEWS: Modified Early Warning Score
NEWS: National Early Warning Score
NEWS-C: A Modified version of the National Early Warning Score for COVID-19 Infected Patient
NEWS2: National Early Warning Score 2
NEWS2 Plus: National Early Warning Score 2 with age and body mass index
qSOFA: Quick Sepsis-related Organ Failure Assessment scores
ROC: Receiver operating characteristic
AUC: Area under the receiver operating characteristic
PPV: Positive predictive value
NPV: Negative predictive value
LR+: Positive likelihood ratio
LR-: Negative likelihood ratio
