# Supplementary material for: Evaluation of physiological severity scores for predicting COVID-19 disease progression: a retrospective study
Source: BMC Infect Dis. 2025 May 26;25:758. doi: 10.1186/s12879-025-11127-7 (PMC12107795; doi:10.1186/s12879-025-11127-7)
Supplement: Supplementary file 1 — Supplementary Material 1. [file 12879_2025_11127_MOESM1_ESM.docx]

**Supplementary information**

**Supplementary Table 1**

**Scoring systems of Physiological Severity Scores.**

The Early Warning Scores were calculated by adding the individual assigned points together. A = alert; CAM, Confusion assessment method (CAM) screening possible for potential delirium; CNS, central nervous system; GCS, Glasgow Coma Scale; P, pain; U, unresponsive; V, voice.

**The National Early Warning Score (NEWS)**

| Physiologic Parameters | 3 | 2 | 1 | 0 | 1 | 2 | 3 |
| --- | --- | --- | --- | --- | --- | --- | --- |
| Respiratory Rate (bpm) | <8 |  | 9-11 | 12-20 |  | 21-24 | ≥25 |
| SpO_2_ Scale (%) | ≤91 | 92-93 | 94-95 | ≥96 |  |  |  |
| Oxygen supplement |  | Oxygen |  | Air |  |  |  |
| Systolic Blood Pressure (mmHg) | ≤90 | 91-100 | 101-110 | 111-219 |  |  | ≥220 |
| Heart Rate (bpm) | ≤40 |  | 41-50 | 51-90 | 91-110 | 111-130 | ≥131 |
| Consciousness |  |  |  | Alert |  |  | VPU |
| Temperature (ºC) | ≤35.0 |  | 35.1-36.0 | 36.1-38.0 | 38.1-39.0 | ≥39.1 |  |

Abbreviations; V, verbal; P, Pain; U, none.

**A Modified version of the National Early Warning Score for COVID-19 Infected Patient (NEWS-C)**

| Physiologic Parameters | 3 | 2 | 1 | 0 | 1 | 2 | 3 |
| --- | --- | --- | --- | --- | --- | --- | --- |
| Age (years) |  |  |  | <65 |  |  | ≥65 |
| Respiratory Rate (bpm) | <8 |  | 9-11 | 12-20 |  | 21-24 | ≥25 |
| SpO_2_ Scale (%) | ≤91 | 92-93 | 94-95 | ≥96 |  |  |  |
| Oxygen supplement |  | Oxygen |  | Air |  |  |  |
| Systolic Blood Pressure (mmHg) | ≤90 | 91-100 | 101-110 | 111-219 |  |  | ≥220 |
| Heart Rate (bpm) | ≤40 |  | 41-50 | 51-90 | 91-110 | 111-130 | ≥131 |
| Consciousness |  |  |  | Alert |  |  | VPU |
| Temperature (ºC) | ≤35.0 |  | 35.1-36.0 | 36.1-38.0 | 38.1-39.0 | ≥39.1 |  |

Abbreviations; V, verbal; P, Pain; U, none.

**National Early Warning Score 2 (NEWS2)**

| Physiologic Parameters | 3 | 2 | 1 | 0 | 1 | 2 | 3 |
| --- | --- | --- | --- | --- | --- | --- | --- |
| Respiratory Rate (bpm) | <8 |  | 9-11 | 12-20 |  | 21-24 | ≥25 |
| SpO_2_ Scale 1 (%) | ≤91 | 92-93 | 94-95 | ≥96 |  |  |  |
| SpO_2_ Scale 2 (%) | ≤83 | 84-85 | 86-87 | 88-92  ≥93 on air | 93-94  on oxygen | 95-96 on oxygen | ≥97  on oxygen |
| Oxygen supplement |  | Oxygen |  | Air |  |  |  |
| Systolic Blood Pressure (mmHg) | ≤90 | 91-100 | 101-110 | 111-219 |  |  | ≥220 |
| Heart Rate (bpm) | ≤40 |  | 41-50 | 51-90 | 91-110 | 111-130 | ≥131 |
| Consciousness |  |  |  | Alert |  |  | VPU |
| Temperature (ºC) | ≤35.0 |  | 35.1-36.0 | 36.1-38.0 | 38.1-39.0 | ≥39.1 |  |

Abbreviations; V, verbal; P, Pain; U, none.

**National Early Warning Score 2 with age and body mass index (NEWS2 Plus)**

| Physiologic Parameters | 3 | 2 | 1 | 0 | 1 | 2 | 3 | 4 |
| --- | --- | --- | --- | --- | --- | --- | --- | --- |
| Age (years) |  |  |  | <40 |  |  | 40-59 | ≥60 |
| Body Mass Index (kg/m^2^) |  |  |  | <24.9 |  | 25.0-29.9 | ≥30 |  |
| Respiratory Rate (bpm) | <8 |  | 9-11 | 12-20 |  | 21-24 | ≥25 |  |
| SpO_2_ Scale 1 (%) | ≤91 | 92-93 | 94-95 | ≥96 |  |  |  |  |
| SpO_2_ Scale 2 (%) | ≤83 | 84-85 | 86-87 | 88-92  ≥93 on air | 93-94  on oxygen | 95-96 on oxygen | ≥97  on oxygen |  |
| Oxygen supplement |  | Oxygen |  | Air |  |  |  |  |
| Systolic Blood Pressure (mmHg) | ≤90 | 91-100 | 101-110 | 111-219 |  |  | ≥220 |  |
| Heart Rate (bpm) | ≤40 |  | 41-50 | 51-90 | 91-110 | 111-130 | ≥131 |  |
| Consciousness |  |  |  | Alert |  |  | VPU |  |
| Temperature (ºC) | ≤35.0 |  | 35.1-36.0 | 36.1-38.0 | 38.1-39.0 | ≥39.1 |  |  |

Abbreviations; V, verbal; P, Pain; U, none.

**Hamilton Early Warning Score (HEWS)**

| Physiologic Parameters | 3 | 2 | 1 | 0 | 1 | 2 | 3 |
| --- | --- | --- | --- | --- | --- | --- | --- |
| Respiratory Rate (bpm) | <8 | 8-13 |  | 14-20 |  | 21-30 | >30 |
| SpO_2_ Scale (%) | <85 |  | 85-92 | >92 |  |  |  |
| Oxygen supplement |  |  |  | Room air | ≤5 L/min |  | >5L/min |
| Systolic Blood Pressure (mmHg) | <71 | 71-90 |  | 91-170 |  | 171-200 | >200 |
| Heart Rate (bpm) |  | ≤40 | 41-50 | 51-100 | 101-110 | 111-130 | >130 |
| CNS Change from Baseline |  | CAM |  | A | V | P | U |
| Temperature (ºC) | ≤35 |  | 35.1-36 | 36.1-37.9 | 38-39 | ≥39.1 |  |

Abbreviations; A, Alert, V, verbal; P, Pain; U, none.

**Modified Early Warning Score (MEWS)**

| Physiologic Parameters | 3 | 2 | 1 | 0 | 1 | 2 | 3 |
| --- | --- | --- | --- | --- | --- | --- | --- |
| Respiratory Rate (bpm) |  | <9 |  | 9-14 | 15-20 | 21-29 | ≥30 |
| Systolic Blood Pressure (mmHg) | ≤70 | 71-80 | 81-100 | 101-199 |  | ≥200 |  |
| Heart Rate (bpm) |  | ≤40 | 41-50 | 51-100 | 101-110 | 111-129 | ≥130 |
| CNS Change from Baseline |  |  |  | A | V | P | U |
| Temperature (ºC) |  | ≤35 |  | 35-38.4 |  | ≥38.5 |  |

Abbreviations; A, Alert, V, verbal; P, Pain; U, none.

**Quick Sepsis-related Organ Failure Assessment (qSOFA)**

| Clinical parameters | 0 | 1 |
| --- | --- | --- |
| Respiratory rate (bpm) | <21 | ≥22 |
| Systolic Blood pressure (mmHg) | >100 | ≤ 100 |
| Altered mentation | GCS =15 | GCS <15 |

**Supplementary Table 2**

**Comparison the cutoff point of physiological severity scores for predicting COVID-19 disease progression.**

| **Physiological Severity scores** | **Cutoff point from Youden index** | **Sensitivity (%)** | **Specificity (%)** | **PPV** | **NPV** | **+LR** | **-LR** |
| --- | --- | --- | --- | --- | --- | --- | --- |
| NEWS2 Plus | 4.5 | 83.3 | 61.5 | 31.1 | 94.7 | 2.16 | 0.27 |
| NEWS-C | 2.5 | 63.3 | 68.1 | 29.2 | 89.9 | 1.98 | 0.54 |
| NEWS | 2.5 | 36.7 | 86.1 | 35.5 | 86.7 | 2.64 | 0.74 |
| HEWS | 0.5 | 63.3 | 60.4 | 25.0 | 88.8 | 1.60 | 0.61 |
| NEWS2 | 2.5 | 31.7 | 87.9 | 35.2 | 86.1 | 2.61 | 0.78 |
| MEWS | 1.5 | 53.3 | 67.7 | 25.6 | 87.4 | 1.65 | 0.69 |
| qSOFA | 0.5 | 30.0 | 88.2 | 34.6 | 85.8 | 2.54 | 0.79 |
